# Genome-wide association and Mendelian randomization analyses of placental efficiency and piglet birth weight in Danish Large White pigs

**DOI:** 10.5713/ab.250992

**Published:** 2026-04-02

**Authors:** Shi Xin Yu, Sheng Di Cui, Dong Chen, Zhen Jian Zhao, Patrick Kofi Makafui Tecku, Jia Miao Chen, Run Jie Huang, Yao Xi Zhou, Guo Qing Tang

**Affiliations:** 1 State Key Laboratory of Swine and Poultry Breeding Industry, College of Animal Science and Technology, Sichuan Agricultural University, Chengdu, China; 2 Key Laboratory of Livestock and Poultry Multi-omics, Ministry of Agriculture and Rural Affairs, College of Animal Science and Technology, Sichuan Agricultural University, Chengdu, China; 3 Farm Animal Germplasm Resources and Biotech Breeding Key Laboratory of Sichuan Province, College of Animal Science and Technology, Sichuan Agricultural University, Chengdu, China; 4 Key Laboratory of Agricultural Bioinformatics, Ministry of Education, Sichuan Agricultural University, Chengdu, China

**Keywords:** Birth Weight, Large White, Litter Size, Placental Area, Placental Efficiency

## Abstract

**Objective:**

Modern swine breeding has been shifted from maximizing productivity to improving quality for better efficiency. A key goal is to balance litter size with piglet birth weight (BW). This study focused on Danish Large White pigs to evaluate a novel placental efficiency (PEA) index based on placental area (PA) as a biologically relevant and measurable trait for breeding programs.

**Methods:**

The study integrated genome-wide association studies and Mendelian randomization to explore the genetic relationship and potential causal direction between the proposed PEA index and piglet BW. The investigation used data from 113 piglets.

**Results:**

Analysis revealed a significant positive causal effect of PEA on BW. Nine significant single nucleotide polymorphisms and eight candidate genes (*MARVELD3*, *CMTR2*, *TLE7*, *CHST4*, *TAT*, *ZNF23*, *CALB2*, *PHLPP2*) were identified. Functional enrichment analysis indicated that these genes synergistically regulate placental function: *TAT* is involved in amino acid metabolism and energy homeostasis; *CALB2* may influence trophoblast signaling and placental vascular function via calcium regulation; *CHST4* participates in extracellular matrix modification at the maternal-fetal interface; *MARVELD3* contributes to placental barrier maintenance; *CMTR2* and *TLE7* are involved in RNA modification and transcriptional regulation; *ZNF23* may regulate cell cycle and differentiation; and *PHLPP2* influences cell survival and metabolic adaptation via AKT signaling. These network regulates placental endothelial activity, optimizes nutrient allocation, and positively impacts BW.

**Conclusion:**

This study clarifies, from a genetic perspective, the causal relationship between PEA and BW. The proposed PEA index is presented as a reliable tool for evaluating placental spatial utilization efficiency in polytocous animal breeding. It offers new genetic insights and a potential approach for improving litter viability.

## INTRODUCTION

Reproductive performance is one of the most critical economic traits in swine production. In recent years, integrated breeding strategies based on genomic selection have significantly increased litter size in sows [1]. However, this increase in litter size has been accompanied by challenges, such as reduced average birth weight (BW) and poorer within-litter uniformity [2]. Specifically, the heterogeneity in piglet BW has become more pronounced, leading to a greater number of weak piglets per litter with a BW below 1.0 kg. These low-birth-weight piglets have increased disease susceptibility during later rearing stages, leading to higher pre-weaning mortality rates [3]. Further studies reveal that increased litter size causes a relative insufficiency of intrauterine space, ultimately contributing to placental insufficiency and fetal intrauterine growth restriction (IUGR) [4]. The placenta is a key organ involved in fetal growth during gestation and plays an indispensable role in preventing maternal immune rejection, facilitating materno-fetal exchange, and secreting pregnancy-associated hormones and growth factors. It is essential for maintaining normal fetal development and maternal physiological homeostasis. Placental efficiency (PE) has traditionally been defined as the ratio of fetal weight to placental weight (PW). However, this metric has certain limitations in polytocous species, such as swine. Given that uterine capacity is a key maternal constraint in polytocous pregnancies, the placental area (PA) provides a more direct measure of the spatial occupancy within the uterus compared to PW. Some studies have proposed defining PE as the fetal weight produced per unit of placental exchange area. Nevertheless, this approach is seldom adopted in practice due to the time-consuming, costly, and technically demanding nature of the required measurements. In contrast, PA, as an intuitive and stable morphological trait, is more suitable for phenotypic data collection in genetic breeding research. Therefore, this study employs the ratio of BW to PA to represent PE, aiming to quantify the level of fetal growth supported per unit of PA and thus more accurately reflect the efficiency of substance transport at the maternal-fetal interface.

With the advancement of genomic technologies, genome-wide association studies (GWAS) have become a crucial tool for dissecting the genetic architecture of complex quantitative traits [5–7]. Although previous studies have conducted GWAS on PW [8] and fetal birth weight [9], the genetic mechanisms underlying novel efficiency traits based on PA remain poorly understood. Therefore, this study performed a GWAS on PE in 113 Danish Large White pigs to identify associated single nucleotide polymorphisms (SNPs) and potential candidate genes. Furthermore, Mendelian randomization (MR) was employed to investigate the causal relationship between PE and piglet birth weight. This research aims to elucidate the genetic mechanisms by which placental spatial resource allocation influences fetal growth, thereby providing a theoretical basis for improving the litter size of healthy piglets.

## MATERIALS AND METHODS

### Experimental animals and sample collection

The experimental subjects were newborn piglets from purebred Danish Large White sows (parities 2 to 4), all provided by a commercial farm in Wangjiang, Sichuan Province, China. One or two clinically healthy piglets per litter with no congenital disabilities or physical injuries were selected, resulting in the collection of 113 placental samples and the recording of corresponding trait measurements.

### Collection of placental and umbilical cord tissues

During farrowing, the umbilical cord was ligated approximately 5 cm from the fetal end using sterile, labeled suture. A second suture with an identical label was placed 5–8 cm distal to the first ligation point. The cord was then transected between the two sutures, allowing the labeled segment to retract into the uterus. Each piglet was marked with a corresponding number using a wax pencil. After the marking dried, the piglet was weighed, and its BW was recorded.

Upon expulsion, each placenta was immediately processed by removing the attached amnion, any necrotic areas of avascular chorioallantois, and residual umbilical structures. A tissue sample was collected from within a 3 cm radius of the umbilical cord insertion site at the placental center. This sample was rinsed with phosphate-buffered saline (PBS), flash-frozen in liquid nitrogen, and stored for subsequent sequencing. A separate placental tissue block (approximately 2 cm×2 cm) was fixed in 4% paraformaldehyde solution, sealed, and preserved for subsequent histological sectioning. The placenta was then spread without folds on a grid-lined sulfate paper, and its outline was traced. The PA was estimated by counting the number of 1 cm×1 cm grid squares covered by the tracing, applying the rule of “counting squares over half-covered and disregarding those less than half-covered,” and then multiplying the total count by 2. The PW was measured using an electronic balance. The lengths of both umbilical cord segments (umbilical cord length, UCL) and the diameter of the fetal end of the cord (umbilical cord diameter, UCD) were measured using a vernier caliper.

### Reproductive trait data collection

Detailed pedigree information and reproductive performance were recorded for all Large White sows involved in the trial. The recorded reproductive traits included five key indicators: parity, total number born (TNB), number born alive (NBA), number of healthy live piglets, and litter birth weight (LBW).

### Placental efficienc2y calculation

PE is conventionally defined as the ratio of BW to PW. For clarity, this metric is referred to herein as placental efficiency weight (PEW). In this study, a novel PE index based on PA, designated placental efficiency area (PEA), is introduced. The formula is as follows:


(1) $$PEA=\frac{BW}{PA}$$

### Histomorphological analysis

Following standard histological procedures, placental tissues were fixed in buffered 4% paraformaldehyde for 24 hours. The samples were subsequently dehydrated through a graded ethanol series, cleared in xylene, embedded in paraffin, and sectioned. After deparaffinization and rehydration, the sections were stained with hematoxylin and eosin (H&E) according to standard protocols. The stained sections were mounted and scanned using a light microscope for whole-slide imaging to acquire tissue images for morphological analysis.

Morphometric measurements were performed on the placental sections using CaseViewer v.2.4 software. Ten random fields of view were selected from each placental section. The number of villi and blood vessels within each field (area: 2.7 mm^2^) was counted. The placental villous density (PVD) and vessel density (VD) per unit area were calculated based on these counts.

### Library construction and resequencing

Genomic DNA was extracted from placental tissues using a commercial DNA extraction kit. The integrity of the DNA samples was assessed by agarose gel electrophoresis, and the concentration was accurately quantified using a Qubit 2.0 Fluorometer. After passing stringent quality control, resequencing libraries were constructed with the GenoBaits DNA Library Prep Kit.

Following library preparation, the initial concentration was measured using the Qubit 2.0 Fluorometer, and the effective concentration was precisely quantified via qPCR to ensure the library met sequencing standards. Finally, qualified libraries were sequenced on the Illumina NovaSeq 6000 platform (PE150 mode) to generate raw sequencing data for subsequent analysis.

### Genotyping and quality control

Raw sequencing reads were first filtered using fastp v.0.20.0 [10] to obtain clean reads. The clean reads were then aligned to the pig reference genome (Sscrofa11.1) using BWA-MEM v.0.7.17 [11]. After alignment, duplicate reads were marked and removed using Picard Tools v.3.3.0. Variant calling was performed on the deduplicated SAM files using GATK v.4.1.9 [12] HaplotypeCaller. The resulting gVCF files were genotyped with GATK GenotypeGVCFs, and SNPs were hard-filtered using GATK VariantFiltration with the following criteria: “QD <2.0 || MQ<40.0 || FS>60.0 || SOR>3.0 || MQRankSum<–12.5 || ReadPosRankSum<–8.0”.

Subsequently, vcftools v.0.1.16 [13] was used to extract all SNP loci on the current chromosome, and sites with a quality score≤100 or those marked as low quality were filtered out. Only biallelic SNPs were retained, and multiallelic loci were removed. The corresponding site information files and genotype files were generated. Next, STITCH v.1.7.0 [14] was used for preliminary genotype imputation for each chromosome, with parameters set to K = 10, nGen = 100, and 40 EM iterations.

Finally, bcftools v.1.19 [15] was used to implement stringent quality control, retaining SNPs that met the following criteria: info score>0.98, minor allele frequency (MAF)>0.05, missing rate<0.02, and Hardy–Weinberg equilibrium test p-value>1×10^−6^.

### Genome-wide association analysis

#### Genome-wide association study

For each trait, a linear mixed model was fitted using the lmer function from the lme4 package [16], with sex, parity, and TNB as fixed effects and litter ID as a random effect. The model residuals were extracted as the adjusted trait for subsequent analyses. Genome-wide association analysis was then performed using GEMMA v.0.98.5 [17] with a single-marker mixed linear model (MLM), configured as follows:


(2) y=u+Xb+Sβ+Za+e

*y* is the vector of observed phenotypic values for the trait; *u* is the vector of the overall mean; *b* is the vector of fixed effects, including parity and sex; *β* is the effect of the single-marker SNP; *a* is the vector of random additive genetic effects, assumed to follow a multivariate normal distribution *a*~*N*(0,*A*σ*_a_*^2^), with *A* representing the genomic relationship matrix and σ*_a_*^2^ denoting the additive genetic variance; and *e* is the vector of residual effects. The matrices *X*, *S*, and *Z* are the incidence matrices associating the corresponding effects with the phenotypic observations.

#### Determination of significance thresholds

To control for false positives arising from multiple testing, the Bonferroni correction method was applied to establish genome-wide significance thresholds. The number of SNPs retained after linkage disequilibrium (LD) pruning (using the parameters --indep-pairwise 50 5 0.2) was denoted as N. The genome-wide significance threshold was set at 0.05/N, and the suggestive significance threshold was set at 1/N. The GWAS results were visualized using the CMplot R package [18], generating Manhattan plots and quantile-quantile (Q-Q) plots.

#### Linkage disequilibrium analysis and gene annotation

To analyze the LD patterns among significant SNPs, LDblockShow v.1.40 [19] was used to perform LD block analysis on the region encompassing each significant SNP and its 200 kb upstream and downstream. Genes located within these regions were subsequently extracted as candidate genes. Gene Ontology (GO) and Kyoto Encyclopedia of Genes and Genomes (KEGG) enrichment analyses were conducted on the set of candidate genes using the clusterProfiler v.4.10.1 R package [20]. A significance threshold of p<0.05 was applied to identify statistically significant biological processes and pathways.

### Mendelian randomization analysis

Significant SNPs were clumped using PLINK v.1.90 [21] (with parameters --clump-r2 0.001 and --clump-kb 10000) to identify independent, significant SNPs for use as instrumental variables.

In this study, five complementary methods from the R package TwoSampleMR v.0.6.15 [22] were employed for MR analysis, primarily including inverse variance weighted (IVW), MR-Egger regression, weighted median, simple mode, and weighted mode methods. Horizontal pleiotropy was assessed using the MR-Egger intercept term; the significance of the MR effect was primarily determined based on the results of the IVW method. The remaining four methods served as sensitivity analyses to test the robustness of the results to pleiotropy and heterogeneity. Cochran’s Q test was used to evaluate heterogeneity among instrumental variables, scatter plots were generated to identify potential outliers, and funnel plots were constructed to assess the robustness and homogeneity of the results. The strength of the instrumental variables was evaluated by calculating the F-statistic using the formula:


(3) $$F=\frac{beta^{2}}{se^{2}}$$

## RESULTS

### Phenotypic statistics

Summary statistics were calculated for 15 traits in Danish Large White pigs, including six placental traits (PW, PA, PVD, VD, PEW, PEA), two umbilical cord traits (UCD, UCL), and seven reproductive traits (BW, HLS, NBA, TNB, PSR, HPR, LBW). The coefficients of variation (CV) ranged from 13.00% to 30.73% (Supplement 1).

### Comparison of trait correlations between placental efficiency weight and placental efficiency area

**Figure 1:**
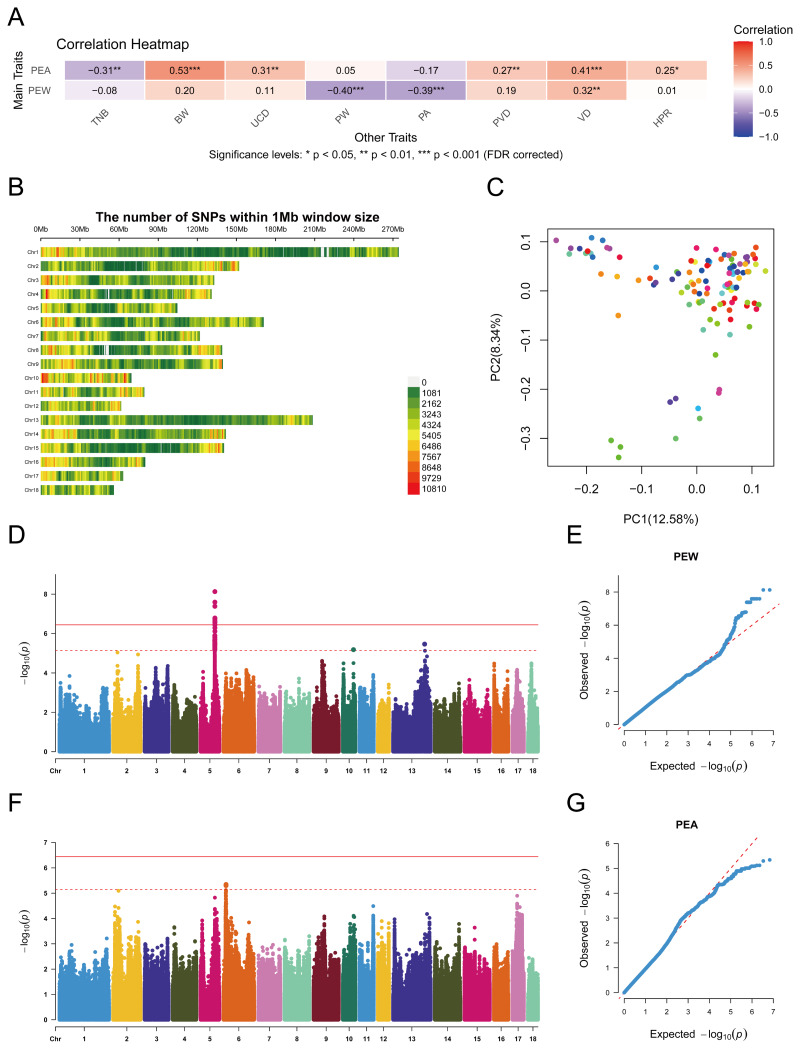
Genetic association analysis for placental efficiency traits. (A) Heatmap of correlations between PEW, PEA and other traits; (B) SNP density plot; (C) Genomic PCA plot of 113 individuals; (D) Manhattan plot of GWAS results for PEW; (E) Q-Q plot of GWAS results for PEW; (F) Manhattan plot of GWAS results for PEA; (G) Q-Q plot of GWAS results for PEA. PEA, placental efficiency area; PEW, placental efficiency weight; TNB, total number born; BW, birth weight; UCD, umbilical cord diameter; PW, placental weight; PA, placental area; PVD, placental villous density; VD, vessel density; HPR, healthy piglet rate; SNP, single nucleotide polymorphism; PCA, principal component analysis; GWAS, genome-wide association studies; Q-Q, quantile-quantile.

To elucidate the biological significance of the two PE indicators (PEW and PEA), this study analyzed their correlations with placental and reproductive traits. The results showed that PEW was highly significantly negatively correlated with PW (r = −0.3953, adjusted p = 0.0001) and PA (r = −0.3870, adjusted p = 0.0001), and highly significantly positively correlated with VD (r = 0.3248, adjusted p = 0.0018). In contrast, PEA was significantly positively correlated with BW (r = 0.5299, adjusted p<0.0001), VD (r = 0.4064, adjusted p = 0.0001), PVD (r = 0.2730, adjusted p = 0.0086), and UCD (r = 0.3092, adjusted p = 0.0025). Regarding reproductive traits, PEA was significantly negatively correlated with TNB (r = −0.3104, adjusted p = 0.0025), and significantly positively correlated with HPR (r = 0.2526, adjusted p = 0.0155) (Figure 1A).

### Genome-wide association study of placental efficiency weight and placental efficiency area

In this study, whole-genome resequencing was performed on 113 Danish Large White pigs. Following a series of steps including reference genome alignment, genotype imputation, data merging, and quality control, a total of 6,761,999 high-quality SNPs were obtained (Figure 1B). Based on these SNPs, a MLM was used to perform genome-wide association analysis for 10 traits, with the kinship matrix (K) and three principal components included as covariates in the model. Principal component analysis (PCA) revealed no evidence of population stratification (Figure 1C). After LD pruning, 138,673 SNPs were retained for subsequent GWAS. Using Bonferroni correction, the genome-wide significance threshold was set at 3.61×10^−6^, and the suggestive threshold at 7.21×10^–6^.

### Candidate gene annotation and enrichment analysis for placental efficiency weight and placental efficiency area

**Figure 2:**
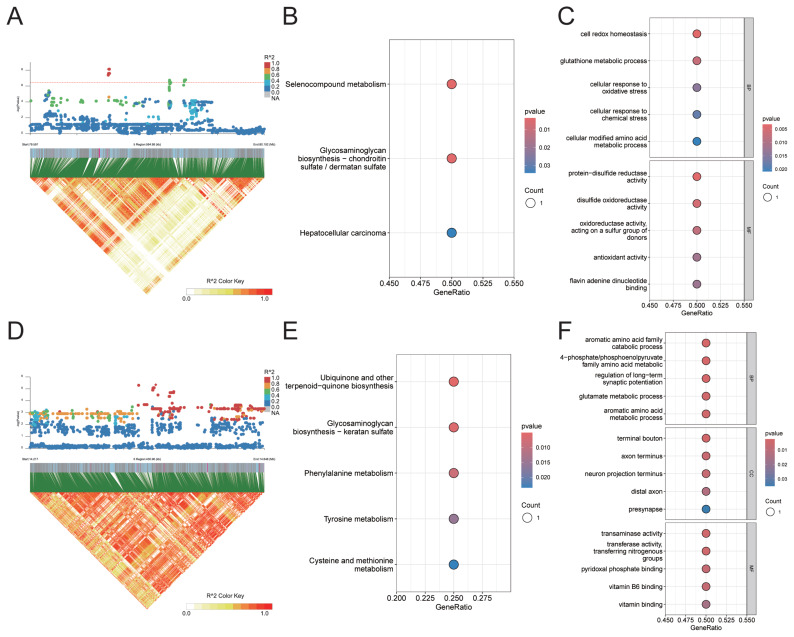
Fine-mapping and enrichment analysis of significant loci for PEW and PEA. (A) LD block plots of the most significant SNPs from the GWAS results for PEW; (B) KEGG enrichment plot of genes annotated from significant SNPs for PEW; (C) GO enrichment plot of genes annotated from significant SNPs for PEW; (D) LD block plots of the most significant SNPs from the GWAS results for PEA; (E) KEGG enrichment plot of genes annotated from significant SNPs for PEA; (F) GO enrichment plot of genes annotated from significant SNPs for PEA. PEW, placental efficiency weight; PEA, placental efficiency area; LD, linkage disequilibrium; SNP, single nucleotide polymorphism; GWAS, genome-wide association studies; KEGG, Kyoto Encyclopedia of Genes and Genomes; GO, Gene Ontology.

For the PEW trait, a total of 29 SNPs reaching the genome-wide significance level were identified and annotated to three candidate genes (Figures 1D, 1E, 2A; Supplement 2). GO enrichment analysis indicated that *TXNRD1* is primarily involved in biological processes such as the maintenance of cellular redox homeostasis, glutathione metabolic process, and cellular response to oxidative and chemical stress. Additionally, this gene plays important roles in sulfur compound metabolism, cellular modified amino acid metabolism, and amide metabolism. At the molecular function level, the protein encoded by *TXNRD1* exhibits protein disulfide reductase activity, disulfide oxidoreductase activity, and oxidoreductase activity acting on sulfur group donors, demonstrating significant antioxidant activity and depending on FAD as a cofactor. KEGG pathway enrichment analysis revealed that *TXNRD1* is mainly involved in selenocompound metabolism and is associated with the pathogenesis of hepatocellular carcinoma. In contrast, *CHST11* was significantly enriched in the glycosaminoglycan biosynthesis pathway, particularly the biosynthesis of chondroitin sulfate/dermatan sulfate (Figures 2B, 2C; Supplement 3).

For the PEA trait, a total of 2 SNPs reaching the suggestive significance level were identified through genome-wide association analysis and were annotated to eight candidate genes: *MARVELD3, CMTR2, TLE7, CHST4, TAT, ZNF23, CALB2*, and *PHLPP2* (Figures 1F, 1G, 2D; Supplement 2). GO enrichment analysis revealed that the *TAT* gene is primarily involved in the catabolism of aromatic amino acids and various amino acid metabolic processes, exhibiting transaminase activity and utilizing pyridoxal phosphate as a cofactor. *CALB2* is involved in the regulation of intracellular calcium homeostasis and the positive regulation of synaptic transmission, with its expression products localized to neuronal presynaptic structures and dendritic compartments, suggesting its role in calcium signal transduction and synaptic function modulation. KEGG pathway enrichment analysis indicated that the *TAT* gene participates in the biosynthesis of ubiquinone and other terpenoid-quinones, as well as metabolic pathways of phenylalanine, tyrosine, cysteine, and methionine. The *CHST4* gene was enriched in the glycosaminoglycan biosynthesis pathway, specifically involved in the synthesis of keratan sulfate (Figures 2E, 2F; Supplement 3).

### Mendelian randomization analysis

This study employed a two-sample MR approach to systematically assess the causal effects of PEW and PEA on BW. Using PLINK’s clump procedure, 1 and 3 significant and independent SNPs were obtained for PEA and PEW, respectively, and served as IVs for the MR analysis.

The MR analysis revealed a significant positive causal effect of PEA on BW. This analysis used a genetic variant associated with PEA as the instrumental variable. Due to the use of only a single instrumental variable, the Wald ratio method was employed for estimation (beta = 1,600.524, p = 0.0139), and the F-statistic was 22.08 (Supplement 4). Due to the limitation of having only one instrumental variable, tests for horizontal pleiotropy and heterogeneity could not be performed.

**Figure 3:**
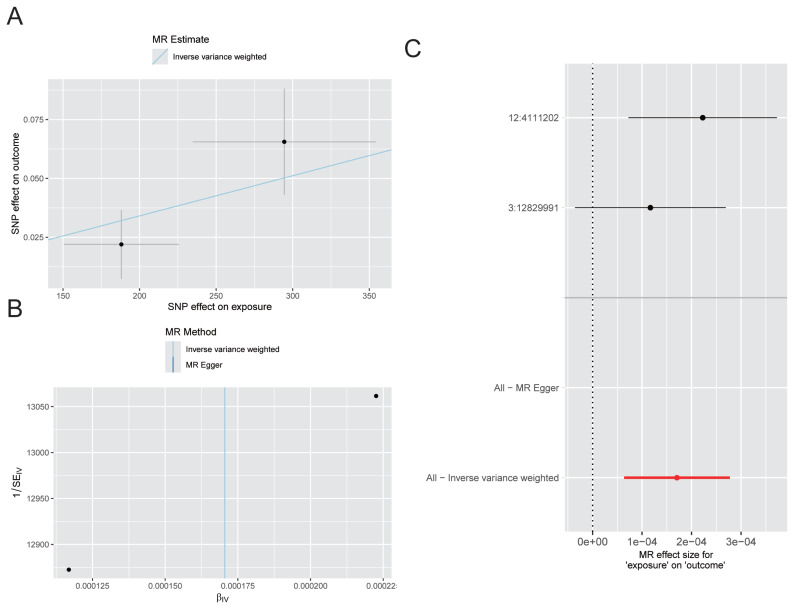
MR results for the effect of BW on PEA. (A) Scatter plot of instrumental variables for the effect of BW on PEA; (B) Funnel plot of instrumental variables for the effect of BW on PEA; (C) Forest plot of instrumental variables for the effect of BW on PEA. MR, Mendelian randomization; SNP, single nucleotide polymorphism; BW, birth weight; PEA, placental efficiency area.

To further verify the reliability of the causal direction, a reverse MR analysis was conducted. The results showed that BW also had a significant positive causal effect on PEA (IVW method, beta = 0.0002, p = 0.0018). The F-statistics for the two instrumental variables used in the reverse analysis ranged from 24.23 to 25.00, indicating no evidence of weak instrument bias. Heterogeneity tests showed no significant heterogeneity among the instrumental variables (IVW method, p = 0.333) (Figure 3; Supplement 4).

**Figure 4:**
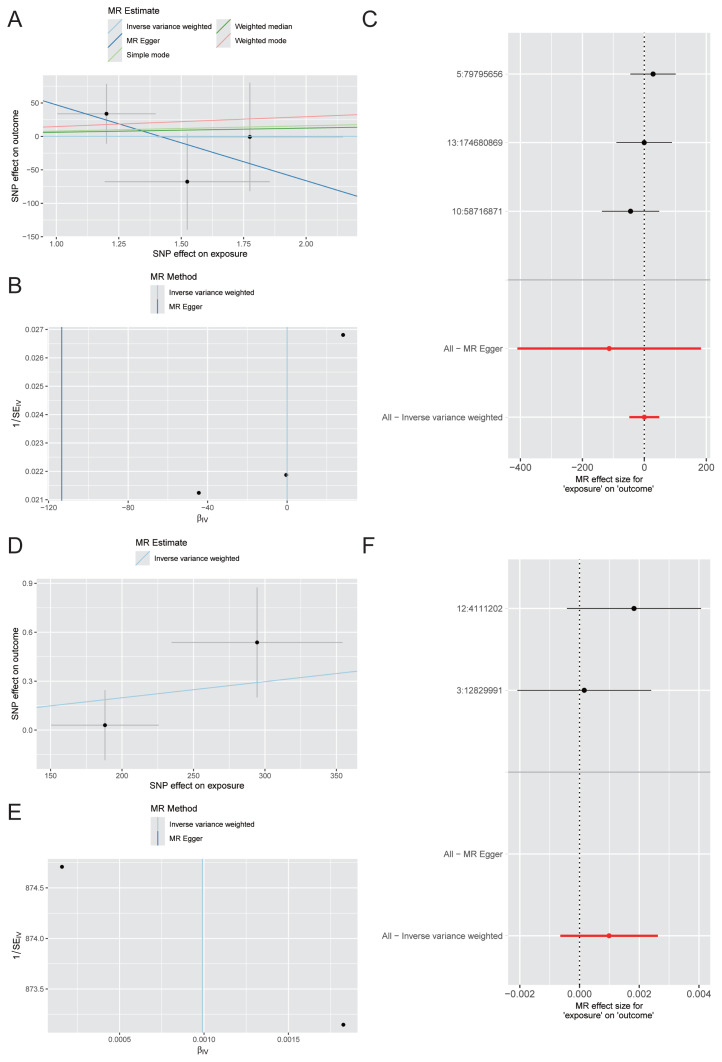
Bidirectional MR analysis between PEW and BW. (A) Scatter plot of instrumental variables for the effect of PEW on BW; (B) Funnel plot of instrumental variables for the effect of PEW on BW; (C) Forest plot of instrumental variables for the effect of PEW on BW; (D) Scatter plot of instrumental variables for the effect of BW on PEW; (E) Funnel plot of instrumental variables for the effect of BW on PEW; (F) Forest plot of instrumental variables for the effect of BW on PEW. MR, Mendelian randomization; SNP, single nucleotide polymorphism; PEW, placental efficiency weight; BW, birth weight.

In contrast, PEW showed no significant causal effect on BW (IVW method, beta = 0.0103, p = 0.9997), with the F-statistics for the three instrumental variables used ranging from 21.26 to 37.16. Reverse MR analysis also did not detect a significant causal effect of BW on PEW (IVW method, beta = 0.0010, p = 0.2345) (Figure 4; Supplement 4).

## DISCUSSION

In modern pig production, genetic selection and reproductive technologies have significantly increased litter size, yet this gain is often accompanied by reduced average piglet BW and lower within-litter uniformity, limiting further improvements in production efficiency. To address this issue, the present study focuses on Danish Large White pigs—a breed with outstanding reproductive performance—and investigates the role of the placenta and umbilical cord as critical structures for materno-fetal nutrient exchange during gestation. While current research on placental traits and BW has mainly centered on IUGR, often employing transcriptomic approaches to explore gene expression differences [23,24], genomic-level investigations remain limited and have largely targeted human populations [8]. Therefore, from a breeding perspective, this study aims to analyze how spatial resource allocation within the placenta affects fetal development, thereby providing a theoretical foundation for genetic strategies to enhance litter uniformity and increase the number of healthy piglets.

This study innovatively proposed a novel PA-based efficiency metric, PEA, and by integrating GWAS and MR analyses, revealed a causal relationship between PEA and BW, ultimately identifying candidate genes associated with PEA. To our knowledge, this represents the first investigation to establish a causal link between PE and piglet BW in Danish Large White pigs.

Comparative analysis of two PE indicators (PEW and PEA) revealed distinct correlation patterns with production traits. PEW showed strong negative correlations with placental size metrics but a highly significant positive correlation with VD, suggesting its improvement stems from functional optimization (e.g., enhanced vascularization) rather than increased placental mass. This finding is consistent with the concept of “PE” proposed in previous studies [25]. Importantly, PEW exhibited no significant correlations with any of the assessed reproductive traits. In contrast, PEA showed significantly positive correlations with BW, VD, PVD, and UCD, with the strongest correlation observed for BW, indicating its significant advantage in promoting fetal growth. Regarding reproductive traits, PEA was negatively associated with TNB and positively associated with HPR, implying a potential trade-off between fetal development quality and litter size under uterine space constraints. Notably, given the sampling limitation (1–2 piglets per litter), these findings should be interpreted as statistical associations, requiring validation through more systematic sampling designs.

GWAS comparison of the two PE metrics revealed distinct genetic architectures: candidate genes for PEW were primarily enriched in pathways related to placental “functional enhancement”. Specifically, *TXNRD1* was implicated in cellular redox homeostasis, glutathione metabolism, and antioxidant stress response [26], suggesting a role in optimizing functional efficiency via redox balance regulation. Additionally, its involvement in selenocompound metabolism and hepatocellular carcinoma pathways implies potential functions in cell proliferation [27]. *CHST11* was significantly enriched in glycosaminoglycan biosynthesis (chondroitin sulfate/dermatan sulfate) [28] and, as a target of TGF-β-like growth factors, may participate in embryonic skeletal development and placental structure construction [29]. Although these genes collectively ensure nutrient supply and functional optimization for individual fetuses, they may also exacerbate developmental disparities among littermates.

In contrast, the candidate genes associated with PEA were more significantly enriched in biological processes such as amino acid metabolism, maintenance of calcium ion homeostasis, regulation of genomic stability, and immune microenvironment balance. These functions help to maintain a relatively stable intrauterine environment within the limited uterine space, thereby enhancing litter uniformity and survival rate, and achieving an overall optimization of population health.

At the metabolic regulation level, GWAS identified distinct candidate genes associated with PEA. *TAT* was significantly enriched in aromatic amino acid catabolism and related metabolic pathways; its encoded protein exhibits transaminase activity, suggesting a role in regulating placental nutrient allocation and embryonic development via amino acid metabolic networks. *CALB2* was primarily involved in cytosolic calcium concentration regulation and calcium homeostasis maintenance. Its expression products are localized in neuronal presynaptic structures, dendrites, and somatodendritic compartments, providing a structural basis for regulating calcium signal transduction and neurotransmitter release [30]. Given that placental trophoblast cells exhibit neuron-like signaling properties, *CALB2* may affect placental vasomotor function and nutrient exchange efficiency by regulating calcium homeostasis. In terms of glycosaminoglycan synthesis, *CHST4* is significantly enriched in the keratan sulfate biosynthesis pathway, participating in extracellular matrix modification and signal transduction regulation, thereby influencing placental microenvironment homeostasis.

At the level of genomic stability, PEA-associated genes synergistically maintain intrauterine homeostasis through multi-dimensional mechanisms. *MARVELD3*, as a tight junction protein, may participate in the maintenance of placental barrier function; *CMTR2* [31] and *TLE7* are involved in mRNA cap methylation modification and transcriptional co-repression regulation, respectively, affecting the stability of gene expression; *ZNF23*, as a zinc finger protein transcription factor, may be involved in cell cycle and differentiation regulation [32]; *PHLPP2*, as a key phosphatase, influences cell survival and metabolic adaptation through negative regulation of the AKT signaling pathway [33]. Together, these genes ensure the precise regulation of placental cell proliferation and differentiation, reduce the risk of developmental abnormalities, and optimize resource allocation by integrating metabolic and signaling networks, thereby promoting the balanced development of the fetal cohort.

We further investigated the causal relationship between PEA and birth weight. The results of the MR analysis showed a significant positive causal effect of PEA on BW (Wald ratio method, beta = 1,600.524, p = 0.0139), and the genetic instrumental variable used had strong statistical power (F-statistic = 22.08). Although limited by the use of a single instrumental variable, which precluded tests for horizontal pleiotropy and heterogeneity, reverse MR analysis revealed that BW also had a significant positive causal effect on PEA (IVW method, beta = 0.0002, p = 0.0018). The instrumental variables used in the reverse analysis had strong power (F-statistic range: 24.23–25.00), and no significant heterogeneity was detected (p = 0.333), suggesting a potential bidirectional promotive relationship between PEA and birth weight.

Integration of GWAS results for PEA identified nine significant SNPs and eight candidate genes (*MARVELD3*, *CMTR2*, *TLE7*, *CHST4*, *TAT*, *ZNF23*, *CALB2*, *PHLPP2*). Functional enrichment analysis revealed that these genes synergistically regulate placental function through multiple biological processes: amino acid metabolism and energy homeostasis (*TAT*), calcium homeostasis and trophoblast signaling (*CALB2*), extracellular matrix modification at the maternal-fetal interface (*CHST4*), placental barrier integrity (*MARVELD3*), RNA modification and transcriptional regulation (*CMTR2*, *TLE7*, *ZNF23*), and cell survival via AKT signaling modulation (*PHLPP2*). Collectively, these mechanisms enhance placental endothelial cell activity, optimize resource allocation and fetal nutrient supply, ultimately exerting a positive effect on birth weight.

These findings align with previous studies highlighting the importance of genomic stability, immune microenvironment balance, trophoblast integrity, and metabolic regulation in maintaining normal pregnancy. Pathological conditions such as preeclampsia and preterm birth are often associated with DNA damage and inflammatory responses, suggesting that dysregulation of the aforementioned mechanisms may lead to adverse pregnancy outcomes. Transcriptomic analyses of placentas affected by IUGR have also revealed significant enrichment of immune and inflammation-related pathways, accompanied by placental villous and capillary dysplasia [23]. Recent genetic studies on BW have further emphasized genes involved in adipose regulation and placental angiogenesis. Building on this, the present study confirms that higher PEA levels may enhance placental structural integrity, metabolic balance, and immune homeostasis, thereby reducing stillbirth rates and the proportion of weak neonates while increasing the number of healthy newborns.

This study proposes a novel comprehensive indicator, PEA, which more effectively reflects the fetal distribution pattern within the limited uterine space of parous animals. Based on this indicator, we employed causal inference methods to dissect the causal relationship between PEA and BW, preliminarily revealing a potential bidirectional promotive effect between the two. This finding provides a new theoretical perspective and potential breeding strategy for improving piglet quality from the perspective of placental development, contributing to an increased proportion of healthy newborn piglets per litter and highlighting the promising application of PEA as a potential breeding target. This aligns with findings from other livestock studies, where selection indices have been effectively used to regulate BW and optimize growth performance [34,35].

However, this study has certain limitations, and the relevant results should be interpreted with caution. First, the modest sample size may limit statistical power and result robustness. Second, in the MR analysis, only one genetic instrument was available for PEA. Although its F-statistic (22.08) met conventional strength standards, the single instrument precluded tests for horizontal pleiotropy and heterogeneity, limiting comprehensive causal assessment. Additionally, potential bias from sample overlap (exposure and outcome data derived from the same population) necessitates validation in larger, independent cohorts. Third, candidate gene identification was primarily based on GWAS statistical inference, representing an exploratory stage without functional experimental validation. The precise biological roles of these genes in placental development and BW regulation require further molecular investigation. Future work will integrate gene expression data for key gene screening, conduct systematic functional validation, and evaluate the practical application of PEA in breeding programs.

Subsequent research will integrate gene expression data to screen for key genes and perform functional validation at the molecular level. Concurrently, we will systematically evaluate the feasibility of incorporating PEA into existing breeding indices and verify its practical effectiveness in increasing the number of healthy newborn piglets per litter and improving within-litter uniformity.

## CONCLUSION

In summary, this study provides the first evidence of a significant positive genetic causal effect of PEA on birth weight, suggesting a potential bidirectional facilitative relationship. Eight candidate genes (*MARVELD3*, *CMTR2*, *TLE7*, *CHST4*, *TAT*, *ZNF23*, *CALB2*, *PHLPP2*) were identified, highlighting the roles of amino acid metabolism, calcium homeostasis, extracellular matrix modification, and transcriptional regulation in optimizing placental function. The PEA indicator demonstrates promising potential for breeding applications, offering a novel theoretical basis for improving piglet quality, increasing healthy neonates per litter, and enhancing BW through genetic approaches.

## Data Availability

Upon reasonable request, the datasets of this study can be available from the corresponding author.

## SUPPLEMENTARY MATERIAL

**Supplement 1**. Statistical information of traits.

**Supplement 2**. Summary of GWAS results for PEA and PEW.

**Supplement 3**. Enrichment results of GWAS-annotated genes.

**Supplement 4**. Summary results of Mendelian randomization.
