# Supplementary material for: Genome-wide association and Mendelian randomization analyses of placental efficiency and piglet birth weight in Danish Large White pigs
Source: Anim Biosci. 2026 Apr 2;39(7):250992. doi: 10.5713/ab.250992 (PMC13353116; doi:10.5713/ab.250992)
Supplement: Supplementary file 1 [file ab-250992-Supplementary-1.pdf]

Supplement 1. Statistical information of traits

Traits	N	Min	Max	Mean	SD	CV
BW, g	113.00	555.00	2128.50	1242.08	340.22	27.39
UCD, cm	113.00	0.63	1.28	1.00	0.13	13.22
UCL, cm	113.00	32.54	74.06	46.72	7.00	14.99
PW, g	113.00	65.00	270.00	143.75	44.18	30.73
PA, cm2	113.00	1062.00	3100.00	2008.97	475.23	23.66
PEW, %	113.00	6.48	13.90	8.84	1.64	18.58
PEA, g/cm2	113.00	0.33	0.96	0.61	0.12	20.24
PVD, mm2	113.00	1.96	5.00	3.78	0.72	18.92
VD, mm2	113.00	3.00	7.93	4.91	0.98	20.02
HLS, N	113.00	5.00	22.00	15.04	3.49	23.21
NBA, N	113.00	5.00	24.00	16.83	4.25	25.27
TNB, N	113.00	5.00	29.00	19.19	4.94	25.74
PSR, %	113.00	0.24	1.00	0.89	0.12	13.00
HPR, %	113.00	0.24	1.00	0.81	0.15	18.23
LBW, kg	113.00	5.94	30.50	21.49	4.41	20.50

Note:BW = Birth Weight of piglets; UCD = Umbilical Cord Diameter; UCL = Umbilical Cord Length; PW = Placental Weight; PA = Placental Area; PEW = Placental Efficiency (%); PEA = Placental Efficiency (g/cm²); PVD = Placental Villus Density; VD = Placental Vascular Density; HLS = Number of Healthy Piglets; NBA = Number of Born Alive; TNB = Total Number Born; PSR = Piglet Survival Rate; HPR = Healthy Piglet Rate; LBW = Litter Birth Weight; N = Number; Min = Minimum Value; Max = Maximum Value; DS = Standard Deviation; CV = Coefficient of Variation
