# Supplementary material for: Genome-wide association and Mendelian randomization analyses of placental efficiency and piglet birth weight in Danish Large White pigs
Source: Anim Biosci. 2026 Apr 2;39(7):250992. doi: 10.5713/ab.250992 (PMC13353116; doi:10.5713/ab.250992)
Supplement: Supplementary file 2 [file ab-250992-Supplementary-2.pdf]

Supplement 2. Summary of GWAS results for PEA and PEW

Traits	Chr	Region	nsnps	top snp	*P*	Genes
PEW	5	79795206-79991666	29	5:79795656	7.43E-09	*TXNRD1, SLC41A2, CHST11*
PEA	6	14417164-14448632	2	6:14448632	4.54E-06	*MARVELD3, CMTR2, TLE7, CHST4, TAT, ZNF23, CALB2, PHLPP2*
