# Supplementary material for: Genome-wide association and Mendelian randomization analyses of placental efficiency and piglet birth weight in Danish Large White pigs
Source: Anim Biosci. 2026 Apr 2;39(7):250992. doi: 10.5713/ab.250992 (PMC13353116; doi:10.5713/ab.250992)
Supplement: Supplementary file 3 [file ab-250992-Supplementary-3.pdf]

Supplement 3. Enrichment results of GWAS-annotated genes

PEW-KEGG

category	subcategory	ID	Description	GeneRatio	BgRatio	*P*	geneID	Count
Metabolism	Metabolism of other amino acids	ssc00450	Selenocompound metabolism	1/2	17/9454	0.0036	396681	1
Metabolism	Glycan biosynthesis and metabolism	ssc00532	Glycosaminoglycan biosynthesis - chondroitin sulfate / dermatan sulfate	1/2	21/9454	0.0044	100157186	1
Human Diseases	Cancer: specific types	ssc05225	Hepatocellular carcinoma	1/2	163/9454	0.0342	396681	1

PEW-GO

ID	Description	GeneRatio	BgRatio	*P*	geneID	Count
GO:0045454	cell redox homeostasis	1/2	13/7920	0.0033	396681	1
GO:0006749	glutathione metabolic process	1/2	27/7920	0.0068	396681	1
GO:0034599	cellular response to oxidative stress	1/2	57/7920	0.0143	396681	1
GO:0062197	cellular response to chemical stress	1/2	72/7920	0.0181	396681	1
GO:0006575	cellular modified amino acid metabolic process	1/2	83/7920	0.0209	396681	1
GO:0006979	response to oxidative stress	1/2	100/7920	0.0251	396681	1
GO:0006790	sulfur compound metabolic process	1/2	143/7920	0.0358	396681	1
GO:0043603	amide metabolic process	1/2	186/7920	0.0464	396681	1
GO:0015035	protein-disulfide reductase activity	1/2	15/7988	0.0038	396681	1
GO:0015036	disulfide oxidoreductase activity	1/2	21/7988	0.0053	396681	1
GO:0016667	oxidoreductase activity, acting on a sulfur group of donors	1/2	31/7988	0.0077	396681	1
GO:0016209	antioxidant activity	1/2	47/7988	0.0117	396681	1
GO:0050660	flavin adenine dinucleotide binding	1/2	49/7988	0.0122	396681	1

PEA-KEGG

category	subcategory	ID	Description	GeneRatio	BgRatio	*P*	geneID	Count
Metabolism	Metabolism of cofactors and vitamins	ssc00130	Ubiquinone and other terpenoid-quinone biosynthesis	1/4	12/9454	0.0051	100511756	1
Metabolism	Glycan biosynthesis and metabolism	ssc00533	Glycosaminoglycan biosynthesis - keratan sulfate	1/4	14/9454	0.0059	106510480	1
Metabolism	Amino acid metabolism	ssc00360	Phenylalanine metabolism	1/4	20/9454	0.0084	100511756	1
Metabolism	Amino acid metabolism	ssc00350	Tyrosine metabolism	1/4	37/9454	0.0156	100511756	1
Metabolism	Amino acid metabolism	ssc00270	Cysteine and methionine metabolism	1/4	56/9454	0.0235	100511756	1

PEA-GO

ID	Description	GeneRatio	BgRatio	*P*	geneID	Count
GO:0009074	aromatic amino acid family catabolic process	1/2	10/7920	0.0025	100511756	1
GO:1902221	erythrose 4-phosphate/phosphoenolpyruvate family amino acid metabolic process	1/2	10/7920	0.0025	100511756	1
GO:1900271	regulation of long-term synaptic potentiation	1/2	11/7920	0.0028	100127479	1
GO:0006536	glutamate metabolic process	1/2	15/7920	0.0038	100511756	1
GO:0009072	aromatic amino acid metabolic process	1/2	16/7920	0.0040	100511756	1
GO:0070050	neuron cellular homeostasis	1/2	19/7920	0.0048	100127479	1
GO:0051480	regulation of cytosolic calcium ion concentration	1/2	24/7920	0.0061	100127479	1
GO:0060291	long-term synaptic potentiation	1/2	24/7920	0.0061	100127479	1
GO:0043648	dicarboxylic acid metabolic process	1/2	31/7920	0.0078	100511756	1
GO:0170040	proteinogenic amino acid catabolic process	1/2	33/7920	0.0083	100511756	1
GO:0170035	L-amino acid catabolic process	1/2	34/7920	0.0086	100511756	1
GO:0009064	glutamine family amino acid metabolic process	1/2	35/7920	0.0088	100511756	1
GO:0050806	positive regulation of synaptic transmission	1/2	35/7920	0.0088	100127479	1
GO:1901606	alpha-amino acid catabolic process	1/2	42/7920	0.0106	100511756	1
GO:0009063	amino acid catabolic process	1/2	50/7920	0.0126	100511756	1
GO:0048167	regulation of synaptic plasticity	1/2	51/7920	0.0128	100127479	1
GO:0170039	proteinogenic amino acid metabolic process	1/2	75/7920	0.0189	100511756	1
GO:0170033	L-amino acid metabolic process	1/2	77/7920	0.0194	100511756	1
GO:1901605	alpha-amino acid metabolic process	1/2	94/7920	0.0236	100511756	1
GO:0006874	intracellular calcium ion homeostasis	1/2	105/7920	0.0263	100127479	1
GO:0055074	calcium ion homeostasis	1/2	109/7920	0.0273	100127479	1
GO:0050804	modulation of chemical synaptic transmission	1/2	112/7920	0.0281	100127479	1
GO:0099177	regulation of trans-synaptic signaling	1/2	112/7920	0.0281	100127479	1
GO:0016054	organic acid catabolic process	1/2	113/7920	0.0283	100511756	1
GO:0046395	carboxylic acid catabolic process	1/2	113/7920	0.0283	100511756	1
GO:0006520	amino acid metabolic process	1/2	130/7920	0.0326	100511756	1
GO:0044282	small molecule catabolic process	1/2	148/7920	0.0370	100511756	1
GO:0098771	inorganic ion homeostasis	1/2	191/7920	0.0477	100127479	1
GO:0030003	intracellular monoatomic cation homeostasis	1/2	197/7920	0.0491	100127479	1
GO:0006873	intracellular monoatomic ion homeostasis	1/2	199/7920	0.0496	100127479	1
GO:0043195	terminal bouton	1/2	15/9127	0.0033	100127479	1
GO:0043679	axon terminus	1/2	28/9127	0.0061	100127479	1
GO:0044306	neuron projection terminus	1/2	31/9127	0.0068	100127479	1
GO:0150034	distal axon	1/2	58/9127	0.0127	100127479	1
GO:0098793	presynapse	1/2	161/9127	0.0350	100127479	1
GO:0030425	dendrite	1/2	167/9127	0.0363	100127479	1
GO:0097447	dendritic tree	1/2	167/9127	0.0363	100127479	1
GO:0030424	axon	1/2	170/9127	0.0369	100127479	1
GO:0036477	somatodendritic compartment	1/2	214/9127	0.0463	100127479	1
GO:0008483	transaminase activity	1/2	16/7988	0.0040	100511756	1
GO:0016769	transferase activity, transferring nitrogenous groups	1/2	19/7988	0.0048	100511756	1
GO:0030170	pyridoxal phosphate binding	1/2	30/7988	0.0075	100511756	1
GO:0070279	vitamin B6 binding	1/2	30/7988	0.0075	100511756	1
GO:0019842	vitamin binding	1/2	63/7988	0.0157	100511756	1
