# Supplementary material for: Genome-wide association and Mendelian randomization analyses of placental efficiency and piglet birth weight in Danish Large White pigs
Source: Anim Biosci. 2026 Apr 2;39(7):250992. doi: 10.5713/ab.250992 (PMC13353116; doi:10.5713/ab.250992)
Supplement: Supplementary file 4 [file ab-250992-Supplementary-4.pdf]

Supplement 4. Summary results of Mendelian randomization

MR Results of PEW-BW

method	nsnp	b	se	*P*
MR Egger	3	-113.4144	151.2206	59.0337%
Weighted median	3	6.2037	29.4703	83.3272%
Inverse variance weighted	3	0.0103	24.6324	99.9665%
Simple mode	3	7.9469	38.8356	85.6797%
Weighted mode	3	14.7331	40.0067	74.8001%

Heterogeneity Results of PEW-BW

method	Q	Q_df	Q_pval
MR Egger	0.8838	1	0.3472
Inverse variance weighted	1.4617	2	0.4815

Leave-One-Out Results of PEW-BW

SNP	b	se	*P*
10:58716871	16.7577	28.9041	5.6207E-01
13:174680869	0.1888	35.3473	9.9574E-01
5:79795656	-21.7813	32.8005	5.0666E-01
All	0.0103	24.6324	9.9967E-01

Pleiotropy Test Results of PEW-BW

egger_intercept	se	*P*
160.5959569	211.2507	0.5862

Single-SNP Results of PEW-BW

SNP	b	se	*P*
10:58716871	-44.4232	47.0805	3.4539E-01
13:174680869	-0.4262	45.7232	9.9256E-01
5:79795656	28.1955	37.3031	4.4974E-01
All - Inverse variance weighted	0.0103	24.6324	9.9967E-01
All - MR Egger	-113.4144	151.2206	5.9034E-01

F-statistic of PEW-BW

SNP	F-statistic
10:58716871	21.2617
13:174680869	22.6916
5:79795656	37.1643

MR Results of BW-PEW

method	nsnp	b	se	*P*
Inverse variance weighted	2	0.0010	0.0008	2.3447E-01

Heterogeneity Results of BW-PEW

method	Q	Q_df	Q_pval
Inverse variance weighted	1.0602	1	0.3032

Leave-One-Out Results of BW-PEW

SNP	b	se	*P*
All	0.0010	0.0008	2.3447E-01

Single-SNP Results of BW-PEW

SNP	b	se	*P*
12:4111202	0.0018	0.0011	1.1103E-01
3:12829991	0.0002	0.0011	8.8947E-01
All - Inverse variance weighted	0.0010	0.0008	2.3447E-01

MR Results of BW-PEW

method	nsnp	b	se	*P*
MR Egger	7	2346.5346	578.6437	9.7749E-03
Weighted median	7	1092.0644	214.0540	3.3641E-07
Inverse variance weighted	7	1145.4901	151.9208	4.6979E-14
Simple mode	7	1234.8716	333.2811	1.0026E-02
Weighted mode	7	1207.8901	324.9171	9.8811E-03

Heterogeneity Results of BW-PEW

method	Q	Q_df	Q_pval
MR Egger	2.4468	5	0.7845
Inverse variance weighted	7.0245	6	0.3186

Leave-One-Out Results of BW-PEW

SNP	b	se	*P*
10:60911854	1032.9324	152.1270	1.1218E-11
14:2979938	1138.7254	178.2813	1.6891E-10
17:40351489	1117.2537	178.4968	3.8686E-10
2:36491866	1175.5369	178.8858	4.9831E-11
6:13530050	1228.5735	150.2604	2.9271E-16
6:29302704	1114.3590	175.1724	1.9980E-10
9:26547985	1208.8648	161.7971	7.9294E-14
All	1145.4901	151.9208	4.6979E-14

F-statistic of BW-PEW

SNP	F-statistic
12:4111202	24.2304
3:12829991	24.9990

MR Results of PEA-BW

method	nsnp	b	se	*P*
Wald ratio	1	1600.5239	650.5518	0.0139

Single-SNP Results of PEA-BW

SNP	b	se	*P*
6:14448632	1600.523928	650.5518265	0.013883801

F-statistic of PEA-BW

SNP	F-statistic
6:14448632	22.0843

MR Results of BW-PEA

method	nsnp	b	se	*P*
Inverse variance weighted	2	0.000170481	5.45E-05	0.001769716

Heterogeneity Results of BW-PEA

method	Q	Q_df	Q_pval
Inverse variance weighted	0.939005882	1	0.332533635

Leave-One-Out Results of BW-PEA

SNP	b	se	*P*
All	0.000170481	5.45E-05	0.001769716

Single-SNP Results of BW-PEA

SNP	b	se	*P*
12:4111202	0.000222556	7.66E-05	0.00364991
3:12829991	0.000116864	7.77E-05	0.132496519
All - Inverse variance weighted	0.000170481	5.45E-05	0.001769716

F-statistic of BW-PEA

SNP	F-statistic
12:4111202	24.2304
3:12829991	24.9990
